# Activation-Inactivation Cycling of Rab35 and ARF6 Is Required for Phagocytosis of Zymosan in RAW264 Macrophages

**DOI:** 10.1155/2015/429439

**Published:** 2015-07-01

**Authors:** Youhei Egami, Makoto Fujii, Katsuhisa Kawai, Yurie Ishikawa, Mitsunori Fukuda, Nobukazu Araki

**Affiliations:** ^1^Department of Histology and Cell Biology, School of Medicine, Kagawa University, Miki, Kagawa 761-0793, Japan; ^2^Laboratory of Membrane Trafficking Mechanisms, Department of Developmental Biology and Neurosciences, Graduate School of Life Sciences, Tohoku University, Aobayama, Aoba-ku, Sendai, Miyagi 980-8578, Japan

## Abstract

Phagocytosis of zymosan by phagocytes is a widely used model of microbial recognition by the innate immune system. Live-cell imaging showed that fluorescent protein-fused Rab35 accumulated in the membranes of phagocytic cups and then dissociated from the membranes of newly formed phagosomes. By our novel pull-down assay for Rab35 activity, we found that Rab35 is deactivated immediately after zymosan internalization into the cells. Phagosome formation was inhibited in cells expressing the GDP- or GTP-locked Rab35 mutant. Moreover, the simultaneous expression of ACAP2—a Rab35 effector protein—with GTP-locked Rab35 or the expression of plasma membrane-targeted ACAP2 showed a marked inhibitory effect on phagocytosis through ARF6 inactivation by the GAP activity of ACAP2. ARF6, a substrate for ACAP2, was also localized on the phagocytic cups and dissociated from the membranes of internalized phagosomes. In support of the microscopic observations, ARF6-GTP pull-down experiments showed that ARF6 is transiently activated during phagosome formation. Furthermore, the expression of GDP- or GTP-locked ARF6 mutants also suppresses the uptake of zymosan. These data suggest that the activation-inactivation cycles of Rab35 and ARF6 are required for the uptake of zymosan and that ACAP2 is an important component that links Rab35/ARF6 signaling during phagocytosis of zymosan.

## 1. Introduction

Phagocytosis is a specialized form of endocytosis that allows the ingestion of large particles (>1 *μ*m in diameter) and plays a critical role in innate immunity and tissue remodeling. Professional phagocytes, mainly macrophages and neutrophils, are equipped to ingest foreign particles, invading microorganisms and apoptotic bodies. They further contribute to the resolution of infections and the clearance of senescent or damaged cells. Phagocytosis of a particle is initiated when that particle binds to a specific cell surface receptor, including Fc*γ* receptors (Fc*γ*Rs), complement receptors, scavenger receptors and Toll-like receptors [1].

Zymosan particles derived from* Saccharomyces cerevisiae* cell walls are mainly composed of carbohydrate polymers like *β*-glucan and serve as a model for recognition of microbes by the innate immune system. It has been reported that phagocytosis of zymosan is mediated by the mannose receptor, *β*-glucan receptor dectin-1, complement receptor 3, and Toll-like receptors 2 and 6 [2–8]. Since zymosan particles are recognized by a variety of receptors on macrophages, phagocytosis of zymosan is a complex sequence of events that involves various signaling cascades by these receptors. The precise molecular mechanism underlying the uptake of zymosan particles remains largely unknown. Importantly, phagocytosis of zymosan is accompanied by actin reorganization, which drives the extension of pseudopodia (phagocytic cups) around zymosan particles. It has been shown that the regulation of actin reorganization during phagocytosis of zymosan depends on Rho-family GTPases, such as Rac1 and Cdc42 [9, 10]. Moreover, actin binding proteins such as talin and myosin I are recruited during phagocytosis of zymosan [11]. These molecules coordinately regulate actin polymerization and reorganization to complete phagocytic cup formation and subsequent cup closure (phagosome formation). After internalization of particles, the newly formed phagosomes mature via a series of interactions with endocytic compartments, eventually fusing with lysosomes for particle degradation [10, 11].

Rab proteins form the largest family of monomeric small GTPases that function as molecular switches by cycling between their inactive GDP-bound and active GTP-bound forms. Whereas the inactive GDP-bound forms are thought to be quiescent in the cytoplasm and complexed with their chaperones, Rab-GDP dissociation inhibitors (GDIs), the active GTP-bound forms physically interact with effectors on the membranes and regulate membrane trafficking. To date, a number of Rab GTPases have been shown to be involved in vesicle formation, motility, docking, and fusion [12–15]. Rab35, a member of the Rab GTPase family [14, 16, 17], is localized on the plasma membrane and some endocytic compartments and regulates an endocytic recycling pathway [18]. Besides its roles in regulating receptor-mediated endocytosis, Rab35 has been shown to be involved in phagocytosis of bacteria [19, 20]. Furthermore, we recently demonstrated that Rab35 regulates phagosome formation during Fc*γ*R-mediated phagocytosis in macrophages [21]. The GTP-bound form of Rab35 recruits downstream effectors, such as fascin (an actin-bundling protein) and MICAL-L1 (a tubular endosomal membrane hub) [22–24]. Importantly, ACAP2 (also known as centaurin *β*2) is recruited to the plasma membrane in a Rab35-dependent manner and functions as a GTPase activating protein (GAP) for ADP-ribosylation factor 6 (ARF6) [25]. ARF6 belongs to the ARF family of small GTP-binding proteins [26, 27]. Like all small GTPases, ARF6 cycles between an inactive GDP-bound form and an active GTP-bound form. ARF6 regulates membrane trafficking between the plasma membrane and endosomal compartment [28, 29] and is involved in remodeling of the actin cytoskeleton [30]. It is noteworthy that ARF6 plays an important role in the internalization of several types of bacteria [31, 32]. In the case of* Chlamydia caviae*, ARF6 is activated during bacterial infection and controls bacterial invasion by actin remodeling [32]. Moreover, just like Rab35, ARF6 has been shown to regulate Fc*γ*R-mediated phagocytosis [33]. Altogether, these findings make both Rab35 and ARF6 good candidates for regulating phagocytosis of zymosan, which is involved in various types of receptors.

In the present study, we present evidence that Rab35/ACAP2/ARF6 are crucial components of zymosan internalization and the activation-inactivation cycles of Rab35 and ARF6 are required for this process.

## 2. Materials and Methods

### 2.1. Reagents

Bovine serum albumin (BSA), zymosan, and Dulbecco's modified Eagle's medium (DMEM) were obtained from Sigma Chemical (St. Louis, MO). Fetal bovine serum (FBS) was obtained from BioSolutions International (Melbourne, Australia). Monoclonal mouse anti-GFP antibody (GF200) (Nacalai Tesque) and monoclonal rabbit anti-ARF6 antibody (Cell Signaling Technology) were purchased from commercial sources. The other reagents were purchased from Wako Pure Chemicals (Osaka, Japan) or Nacalai Tesque (Kyoto, Japan), unless otherwise indicated.

### 2.2. Cell Culture

Mouse macrophage RAW264 cells and COS-7 cells were cultured in DMEM supplemented with 10% heat-inactivated fetal bovine serum (FBS), 100 U/mL penicillin and 100 *μ*g/mL streptomycin, as described in the manuals of the cell line bank. RAW264 cells stably expressing Rab35 shRNA or GFP shRNA were maintained in the culture medium supplemented with 5 *μ*g/mL puromycin, as previously described [21]. Before the imaging experiments, the culture medium was replaced with Ringer's buffer (RB) consisting of 155 mM NaCl, 5 mM KCl, 2 mM CaCl_2_, 1 mM MgCl_2_, 2 mM Na_2_HPO_4_, 10 mM glucose, 10 mM HEPES pH 7.2, and 0.5 mg/mL BSA.

### 2.3. DNA Constructs and Transfection

pEGFP-Rab35, pTagRFP-Rab35, pEGFP-Rab35-Q67L, pTagRFP-Rab35-Q67L, pEGFP-Rab35-S22N, pEGFP-ACAP2, pEGFP- ACAP2-ΔANKR, and pEGFP-ACAP2-RQ were described previously [21, 25]. pECFP-ACAP2 and pECFP-ACAP2-RQ were generated by the replacement of GFP with CFP. ARF6-wt-CFP, ARF6-Q67L-CFP, and ARF6-T27N-CFP were kindly donated by Dr. Joel A. Swanson (University of Michigan). pEGFP-Rab11-Q70L was provided by Dr. Marino Zerial (Max Planck Institute) and pEGFP-Rab21-Q78L was a generous gift from Dr. Arwyn T. Jones (Cardiff University). To generate a pECFP-Mem-C1 vector containing the N-terminal 20 amino acids of GAP-43 and ECFP, plasma membrane-targeted ECFP was amplified using primers: 5′-CGGGACTTTCCAAAATGTCGTAAC-3′ and 5′-GAGATCTGAGTCCGGACTTGTACAGCTCGT-3′ with pECFP-Mem (Clontech, Palo Alto, CA) as a template. The PCR products were subcloned into pGEM-T Easy Vector (Promega, Madison, WI), digested using NheI/BspEI restriction enzymes and cloned into the NheI/BspEI restriction sites of pECFP-C1 (Clontech, Palo Alto, CA). To produce pECFP-Mem-ACAP2 and pECFP-Mem-ACAP2-RQ, the BspEI/SalI fragments of pEGFP-ACAP2 and pEGFP-ACAP2-RQ were ligated into the same site of pECFP-Mem-C1. To construct pGEX-4T3-ANKR encoding GST-ANKR, the ankyrin repeat (ANKR) domain (amino acids 587–777) of ACAP2 was amplified using primers: 5′-TCGAATTCTCCTGAAGGAGAAAGGCAAGAGT-3′ and 5′-ACCGTCGACTCAGAACTTCTGTGAATCTTGCTGGA-3′ with pEGFP-ACAP2 as a template. The fragments were cloned into the EcoRI/SalI sites of pGEX4T-3 (Amersham Life Science, Arlington Heights, IL). pGEX-4T3-GGA3 encoding GST-GGA3 was constructed by PCR amplification of mouse cDNAs. The primers used were as follows: 5′-TCGAATTCTATGGCGGAGGCGGAAGGG-3′ and 5′-ACCGTCGACTCAGTCAGGCATGGTTGAGGT-3′. The fragment was cloned into the EcoRI/SalI restriction sites of pGEX4T-3. All constructs were verified by sequencing prior to use. The RAW264 cells were transfected by the Neon Transfection System (Invitrogen, Carlsbad, CA) according to the manufacturer's instructions. The transfected cells were seeded onto 25 mm coverslips or into 60 mm dishes and cultured in growth medium. COS-7 cells were transfected by electroporation. The cells were suspended at ~10^7^ cells/mL in growth medium. Four hundred *μ*L of the cell suspension was mixed with 5 *μ*g of plasmid DNA in a 4 mm gap electroporation cuvette. Electroporation was performed using an ECM630 Electroporation System (BTX Harvard Apparatus, Inc., MA) at 200 V, 1000 *μ*f, and 25 Ω. Cells were then seeded in a 6 cm dish and maintained in the growth medium. All experiments were performed 16–48 hours after transfection.

### 2.4. Protein Expression and Purification

GST, GST-ANKR, and GST-GGA3 proteins were expressed in* Escherichia coli* (BL21(DE3)). Cells grown in LB medium were incubated in the presence of 0.1 mM isopropyl-1-thio-*β*-D-galactopyranoside (15 hours at 25°C). After centrifugation, resulting cell pellets were resuspended in a buffer containing a mixture of protease inhibitors (20 mM Tris-HCl pH 7.5, 1 mM EDTA, 1 mM dithiothreitol, 0.5 mM phenylmethylsulfonyl fluoride, 50 units/mL aprotinin, 2 *μ*g/mL leupeptin, and 2 *μ*g/mL pepstatin A) and subjected to sonication. The cell debris was removed by centrifugation and the resultant supernatant was used as an* E. coli* lysate. Purification of GST fusion proteins to near homogeneity was achieved using Glutathione-Sepharose 4B affinity chromatography (GE Healthcare, Piscataway, NJ). The purity of the samples was at least 80%, as judged by Coomassie Brilliant Blue staining of SDS-polyacrylamide gel electrophoresis (PAGE).

### 2.5. GST Pull-Down Assay and Western Blotting

Cells were washed with ice-cold phosphate-buffer saline (PBS) and suspended in lysis buffer containing 25 mM Tris-HCl (pH 7.2), 150 mM NaCl, 5 mM MgCl_2_, 1% NP-40, 5% glycerol, and protease inhibitor cocktail (Nacalai Tesque, Kyoto, Japan). The cell lysates were briefly sonicated at 4°C and separated from the pellets after centrifugation at 12,100 g for 15 minutes. Protein concentrations were estimated with the BCA protein assay reagent. Glutathione-sepharose beads coupled with GST, GST-ANKR, or GST-GGA3 were incubated for 2 hours at 4°C with 500 *μ*g of the cell lysates. After washing the beads four times with lysis buffer, proteins bound to the beads were analyzed by 10% or 12.5% SDS-PAGE followed by western blotting. The samples were subjected to SDS-PAGE and transferred to the PVDF membrane (Bio-Rad, Richmond, CA). Western blotting was carried out using a polyvinylidene difluoride (PVDF) membrane and the ECL Prime Western Blotting Detection System (GE Healthcare, Piscataway, NJ). The membrane was blocked with 5% nonfat dry milk in PBS containing 0.1% Tween 20 for 30 minutes at room temperature and probed with primary antibody at 4°C overnight. After washing, the membrane was incubated with horseradish conjugated with anti-rabbit or anti-mouse secondary antibody (dilution 1 : 10,000) for 2 hours at room temperature, developed using an ECL Prime reagent and exposed to Hyperfilm (GE Healthcare, Piscataway, NJ). The following primary antibodies were used: anti-ARF6 antibody (1 : 1,000) and anti-GFP antibody (1 : 2,000). The intensity of the protein bands was measured using Photoshop CS5 software.

### 2.6. Phagocytosis Assay

Zymosan was suspended in PBS and boiled for 30 minutes. After a brief washing, zymosan was resuspended in PBS (10 mg/mL) and sonicated for several minutes to disperse the particles prior to use. For the quantitative assay of phagocytosis, zymosan was added to adherent RAW264 macrophages. After 30 minutes of incubation with zymosan at 37°C, the cells on the coverslips were fixed with 4% paraformaldehyde. The number of internalized zymosan particles was counted in 50 cells randomly chosen under a phase-contrast and fluorescence microscope. The phagocytic index, that is, the mean number of zymosan particles taken up per cell, was calculated. The index obtained for the transfected cells was divided by the index obtained for the untransfected (control) cells and expressed as a percentage of the control cells.

### 2.7. Live-Cell Imaging and Image Analysis

RAW264 cells were cultured onto 25 mm circular coverslips and assembled in an RB-filled chamber on the thermocontrolled stage (Tokai Hit, Shizuoka, Japan). Phase-contrast and fluorescence images of live cells were sequentially captured using an Axio observer Z1 inverted microscope equipped with a laser scanning unit (LSM700, Zeiss) under the control of ZEN2009 software (Zeiss), as previously described [34]. Time-lapse images of phase-contrast and fluorescence microscopy were taken at 15-second intervals. Line scan analysis was performed using the MetaMorph software. At least four examples were observed in each experiment.

### 2.8. Statistical Analysis

All *p* values were two-tailed and considered significant at *p* < 0.05. All statistical analyses were performed with EZR (Saitama Medical Center, Jichi Medical University, Saitama, Japan), which is a graphical user interface for R (The R Foundation for Statistical Computing, Vienna, Austria) [35]. Precisely, it is a modified version of R commander designed to add statistical functions frequently used in biostatics. One-way analysis of variance (ANOVA) was conducted to compare across groups. ANOVA was followed by Tukey's test. Student's *t*-test was used for specific comparisons of two groups.

## 3. Results

### 3.1. Rab35 Is Recruited to the Phagocytic Cups and Regulates Phagosome Formation during Phagocytosis of Zymosan

**Figure 1:**
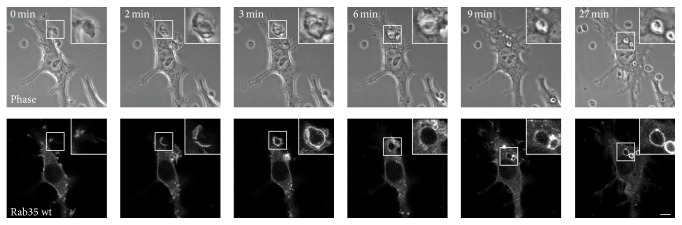
Rab35 is recruited to the phagocytic cups. Live RAW264 macrophages expressing GFP-Rab35 were incubated with zymosan and were observed by confocal laser microscopy. Phase-contrast images are shown (upper panels). The elapsed time is indicated at the top. The binding of zymosan to the cell surface was set as time 0. The insets show higher-magnification images of the indicated regions of the cells. Scale bar: 5 *μ*m.

We previously demonstrated that Rab35 regulates phagosome formation during Fc*γ*R-mediated phagocytosis in macrophages [21]. Therefore, we investigated whether or not Rab35 is also involved in the uptake of zymosan particles. RAW264 macrophages were transfected with GFP-Rab35 wild-type (wt) and observed by confocal laser microscopy. Prior to the beginning of phagocytosis, Rab35 was localized in the cytosol, plasma membrane, and some intracellular vesicles (Figure 1, *t* = 0 min). After the binding of zymosan to the cells, more Rab35 was recruited to the plasma membrane of phagocytic cups (Figure 1, *t* = 2 min and 3 min). Subsequently, Rab35 dissociated from nascent phagosomes (Figure 1, *t* = 6 min). Thereafter, Rab35 again accumulated in maturating phagosomes over an extended period (Figure 1, *t* = 27 min).

**Figure 2:**
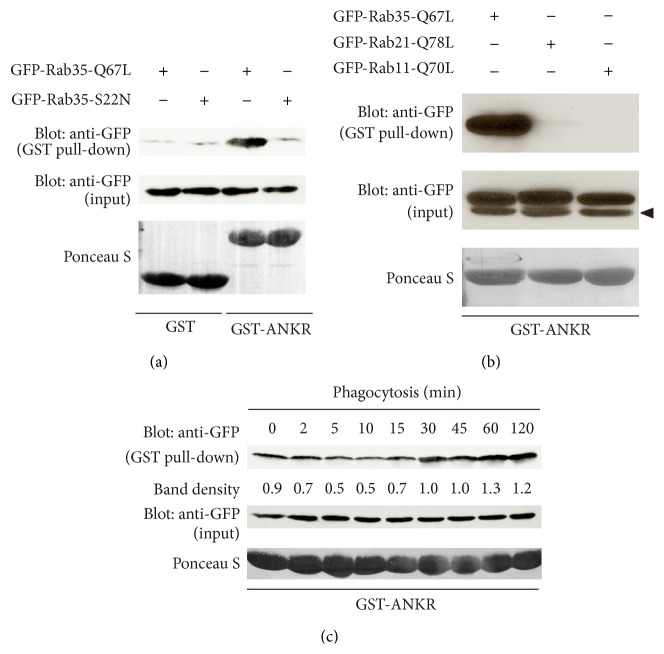
Rab35 is inactivated during phagocytosis of zymosan. (a) GTP-dependent association of GST-ANKR with Rab35-Q67L. COS-7 cell lysates expressing GFP-Rab35-Q67L or GFP-Rab35-S22N were incubated with GST or GST-ANKR. The proteins associated with GST or GST-ANKR were pulled down using glutathione-sepharose beads and analyzed by western blotting (top panel). The middle panel shows aliquots of total cell lysates. (b) Binding specificity of GST-ANKR. Glutathione-sepharose beads coupled with GST-ANKR were incubated with COS-7 cells lysates expressing GFP-Rab35-Q67L, GFP-Rab21-Q78L, or GFP-Rab11-Q70L. Proteins bound to the beads were analyzed by western blotting. The middle panel shows aliquots of total cell lysates. The lower band is a nonspecific band (arrowhead). (c) RAW264 cells expressing GFP-Rab35 were incubated with or without (0 min) zymosan for various times at 37°C. Cell lysates were prepared and incubated with GST-ANKR. The proteins associated with GST-ANKR were pulled down using glutathione-sepharose beads and analyzed by western blotting (top panel). The middle panel shows aliquots of total cell lysates. The density of the protein bands was measured. The relative protein band intensity of GTP-Rab35 was normalized to total Rab35 (GTP-bound plus GDP-bound forms).

The GTP-bound form of Rab35 has been shown to interact with the ankyrin repeat (ANKR) domain of ACAP2 [25]. To quantitatively monitor the activation levels of Rab35 during zymosan phagocytosis, we took advantage of the GTP-dependent interaction of Rab35 with the ANKR domain. First, we prepared a GST fusion containing the ANKR domain of ACAP2 in* Escherichia coli*. Then, the specificity of this recombinant protein was tested using COS-7 cells expressing GTP-bound mutant Rab35-Q67L and GDP-bound mutant Rab35-S22N. As shown in Figure 2(a), only the GTP-bound mutant of Rab35 bound to GST-ANKR, whereas the GDP-bound mutant of Rab35 did not. Moreover, GST-ANKR specifically interacted with GTP-bound mutant Rab35-Q67L but did not interact with GTP-bound mutant Rab21-Q78L or Rab11-Q70L (Figure 2(b)). Based on the data above, we performed the pull-down experiments to measure the activation levels of Rab35 during phagocytosis of zymosan in RAW264 macrophages expressing GFP-Rab35. During phagosome formation, obvious activation of Rab35 was not detected (Figure 2(c), top panel, *t* = 2 min). However, inactivation of Rab35 was observed 5–10 minutes after the addition of zymosan. Subsequently, the GTP-bound form of Rab35 gradually increased over time.

**Figure 3:**
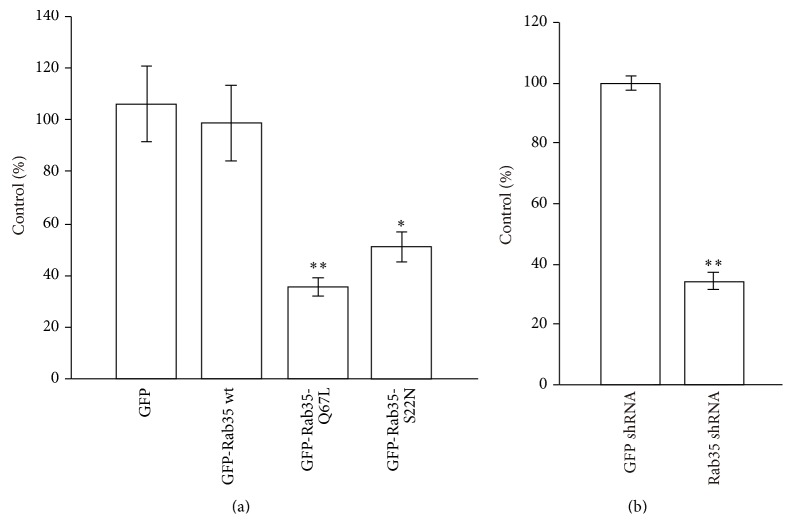
Rab35 regulates phagosome formation. (a) Effect of active and inactive Rab35 mutant expression on zymosan phagocytosis. RAW264 cells expressing each construct indicated were incubated with zymosan and then fixed. The efficiency of phagocytosis was calculated based on 50 transfected and 50 untransfected cells. The results were expressed as a percentage of control (untransfected) cells. The data are expressed as the means ± SEM of four independent experiments. ^*∗*^ *p* < 0.05 and ^*∗∗*^ *p* < 0.01 compared to GFP transfected cells (Student's *t*-test). (b) Effect of Rab35 knockdown on phagocytosis of zymosan. RAW264 cells stably expressing RAB35 shRNA or GFP shRNA were incubated with zymosan and were fixed. The efficiency of phagocytosis was calculated based on 50 cells expressing each Rab35 shRNA and 50 control cells stably transfected with GFP shRNA or empty shRNA vector. The results are expressed as a percentage of control cells stably transfected with empty shRNA plasmid. The data are expressed as the means ± SEM of four independent experiments. ^*∗∗*^ *p* < 0.01 compared to GFP transfected cells (Student's *t*-test).

To confirm the functional contribution of Rab35 to the uptake of zymosan, we examined the effects of Rab35 expression on the phagocytosis of zymosan. We transfected RAW264 cells with GFP-Rab35 wt, GFP-Rab35-Q67L (active form), and GFP-Rab35-S22N (inactive form). Then, a quantitative assay of the phagocytosis of zymosan was performed using cells expressing each Rab35 allele. As shown in Figure 3(a), the expression of either GFP-Rab35-Q67L or GFP-Rab35-S22N led to the inhibition of the uptake of zymosan, in contrast to the GFP control. To determine if endogenous Rab35 is necessary for zymosan phagocytosis, RAW264 cells stably expressing Rab35 shRNA were subjected to a phagocytosis assay [21]. As shown in Figure 3(b), the depletion of endogenous Rab35 led to the inhibition of zymosan phagocytosis. These data suggest that activated Rab35 is not sufficient for the phagocytosis of zymosan, but normal cycling of Rab35 between GTP- and GDP-bound forms is required.

### 3.2. ACAP2 Accumulates at the Phagocytic Cups in a Rab35-Dependent Manner and Inhibits Phagocytosis of Zymosan

**Figure 4:**
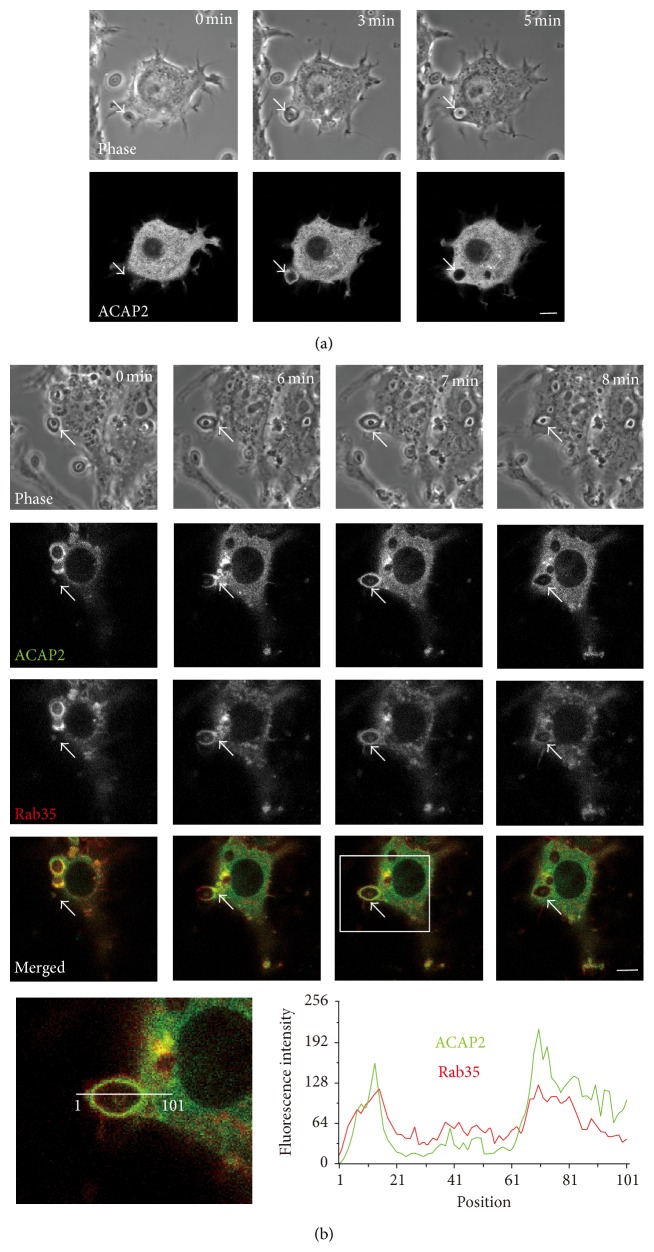
Live-cell imaging of GFP-ACAP2 and Rab35 in RAW264 macrophages during phagocytosis of zymosan. (a) RAW264 cells expressing GFP-ACAP2 were allowed to make contact with zymosan (arrows) and were observed by confocal laser microscopy. Phase-contrast images are shown (top panels). The elapsed time is indicated at the top. The binding of zymosan to the cell surface was set as time 0. Scale bar: 5 *μ*m. (b) Live RAW264 macrophages coexpressing GFP-ACAP2 and TagRFP-Rab35 were incubated with zymosan and were monitored by confocal microscopy. Scale bar: 5 *μ*m. Boxed area is enlarged (bottom left). Line scan analysis using MetaMorph program shows fluorescence intensities of GFP-ACAP2 (green) and TagRFP-Rab35 (red) at the position of the line in the enlarged image.

During Fc*γ*R-mediated phagocytosis, ACAP2, an ARF6-specific GAP (GTPase activating protein), is recruited to phagocytic cups and regulates phagosome formation in a Rab35-dependent manner [21]. Hence, we observed the subcellular localization of ACAP2 during the uptake of zymosan particles. Time-lapse observation of live RAW264 cells expressing GFP-ACAP2 indicated that before the onset of phagocytosis ACAP2 was distributed throughout the cytosol, as previously shown [25, 36]. After the binding of zymosan to the cells, a small amount of ACAP2 was localized to the membrane of phagocytic cup (Figure 4(a), *t* = 3 min). Subsequently, ACAP2 was dissociated from nascent phagosomes. To further examine whether or not ACAP2 and Rab35 are colocalized in forming phagosomes during phagocytosis of zymosan, RAW264 cells were cotransfected with GFP-ACAP2 and TagRFP-Rab35 and observed by confocal microscopy. Time-lapse observation and line scan analysis revealed that both proteins were colocalized at the membrane of phagocytic cups (Figure 4(b), *t* = 6 min and 7 min) and then dissociated from nascent phagosomes (Figure 4(b), *t* = 8 min). It is important to note that the coexpression of Rab35 caused ACAP2 to accumulate at the membranes of phagocytic cups (Figure 4(a), *t* = 3 min; Figure 4(b), *t* = 7 min).

**Figure 5:**
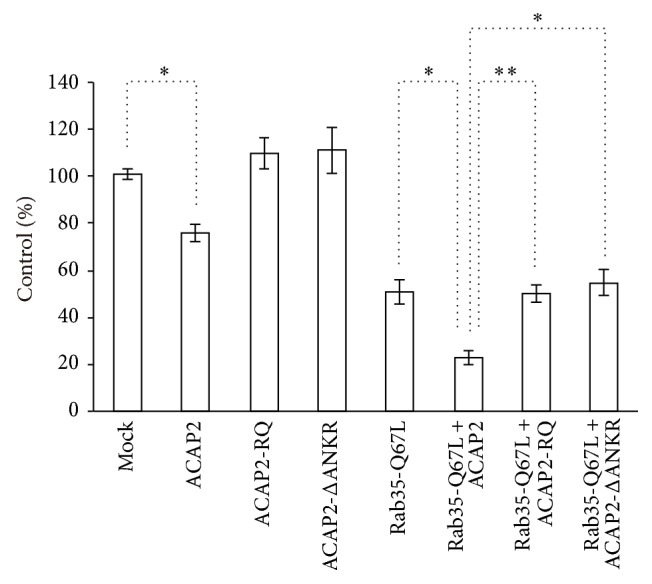
Effects of ACAP2 and/or Rab35 expression on phagocytosis of zymosan. RAW264 macrophages expressing GFP-ACAP2 constructs and/or TagRFP-Rab35-Q67L were incubated with zymosan at 37°C for 30 minutes and were fixed. The efficiency of phagocytosis was calculated based on 50 transfected and 50 untransfected cells. The results were expressed as a percentage of control (untransfected) cells. Data shown are the mean ± SEM of ten independent experiments. Mock indicates cells transfected with GFP and TagRFP. One-way ANOVA followed by Tukey's test was used for statistical analysis (^*∗*^ *p* < 0.05, ^*∗∗*^ *p* < 0.01).

We next examined the effects of the expression of ACAP2 and Rab35 proteins on the uptake of zymosan particles. In a quantitative assay of zymosan phagocytosis (Figure 5), the expression of GFP-ACAP2 reduced the efficiency of phagocytosis slightly. The expression of ARF6-GAP defective mutant of ACAP2 (ACAP2-RQ) or Rab35 binding defective mutant of ACAP2 (ACAP2-ΔANKR) had less effect on the uptake of zymosan. Interestingly, the efficiency of phagocytosis in the cells coexpressing ACAP2 and Rab35-Q67L was significantly lower than that in cells coexpressing ACAP2-RQ or ACAP2-ΔANKR with Rab35-Q67L. These data suggest that the inhibitory effect of ACAP2 on the phagocytosis of zymosan requires GAP activity of ACAP2 toward ARF6 and binding of ACAP2 to Rab35.

### 3.3. ACAP2 Targeted to the Plasma Membrane Inhibits Phagosome Formation through ARF6 Inactivation

**Figure 6:**
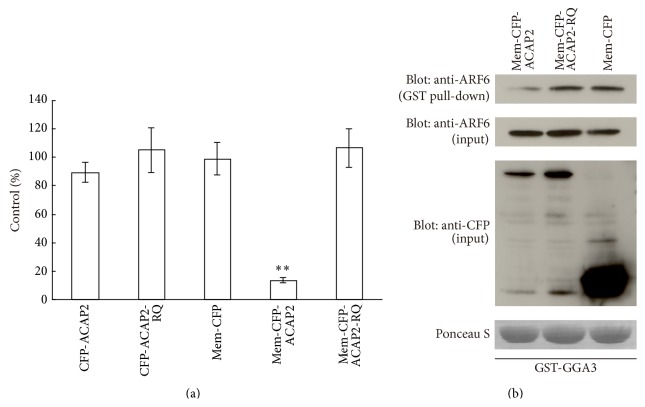
Plasma membrane-targeted ACAP2 inhibits zymosan uptake through ARF6 inactivation. (a) Quantification of zymosan phagocytosis in cells expressing several CFP-ACAP2 mutants was conducted. The number of formed phagosomes was counted under the microscope. The efficiency of phagocytosis was calculated based on 50 transfected and 50 untransfected cells. The results are expressed as a percentage of control (untransfected) cells. The data are means ± SEM of three independent experiments. It is of note that the expression of plasma membrane-targeted ACAP2 dramatically reduces phagosome formation. Student's *t*-test was used for statistical analysis. ^*∗∗*^ *p* < 0.001 compared to Mem-CFP. (b) Levels of endogenous ARF6 activation were determined in RAW264 cells expressing Mem-CFP-ACAP2 or Mem-CFP-ACAP2-RQ. Cell lysates were prepared and incubated with GST-GGA3. The proteins associated with GST-GGA3 were pulled down using glutathione-sepharose beads. GTP-ARF6 (top panel), total ARF6 (second panel), and CFP fusion proteins (third panel) were detected by immunoblotting. GST-GGA3 was stained with Ponceau S (bottom panel). Note that the expression of plasma membrane-targeted ACAP2 impairs ARF6 activation. Similar results were obtained from three independent experiments.

To investigate the relationship between plasma membrane localization of ACAP2 and its ability to inhibit phagocytosis, both ACAP2 wt and ACAP2-RQ were modified by the addition of the palmitoylation sequence to the amino-terminal end, which is sufficient for plasma membrane association [37]. First, RAW264 cells were transfected with these plasma membrane-targeted ACAP2 constructs (Mem-CFP-ACAP2 and Mem-CFP-ACAP2-RQ) and were observed by confocal laser microscopy. As expected, both proteins showed clear plasma membrane localization (data not shown). Then, we evaluated the effect of plasma membrane-targeted ACAP2 expression on the ingestion of zymosan.  Figure 6(a) revealed that expression of Mem-CFP-ACAP2 led to the inhibition of phagocytosis of zymosan, whereas Mem-CFP-ACAP2-RQ expression did not. To determine if the inhibitory effect of Mem-CFP-ACAP2 expression on phagocytosis is caused by inactivation of ARF6, we transfected RAW264 cells with Mem-CFP-ACAP2 and monitored the activation levels of endogenous ARF6. GST pull-down experiments showed that the activation of ARF6 was severely impaired in cells expressing Mem-CFP-ACAP2 compared with those expressing Mem-CFP-ACAP2-RQ and Mem-CFP (Figure 6(b)). Overall, it is suggested that plasma membrane-targeted ACAP2 suppresses phagocytosis of zymosan via ARF6 inactivation.

### 3.4. ARF6 Is Recruited to the Phagocytic Cups and Regulates Phagocytosis of Zymosan

**Figure 7:**
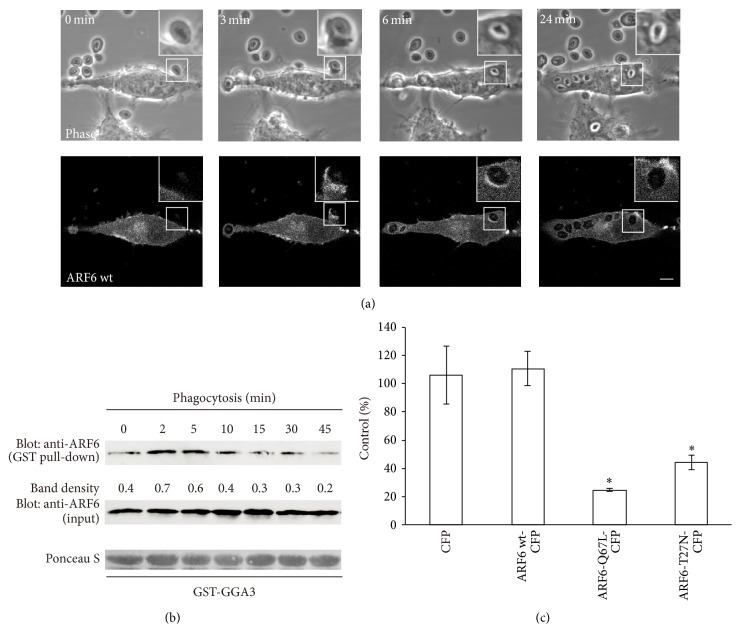
ARF6 is transiently activated and regulates uptake of zymosan particles. (a) Live-cell imaging of RAW264 macrophages expressing ARF6-wt-CFP during the phagocytosis of zymosan. The elapsed time is indicated at the top. The insets show higher-magnification images of the indicated regions of the cells. Phase-contrast images are shown (upper panels). Scale bar: 5 *μ*m. (b) RAW264 cells were incubated with zymosan for various times. Cell lysates were incubated with GST-GGA3. The proteins associated with GST-GGA3 were pulled down using glutathione-sepharose beads and analyzed by western blotting (top panel). The middle panel shows aliquots of total lysates. The density of the protein bands was measured. The relative protein band intensity of GTP-ARF6 was normalized to total ARF6 (GTP-bound plus GDP-bound forms). (c) RAW264 cells transiently expressing CFP, ARF6-wt-CFP, ARF6-Q67L-CFP, or ARF6-T27N-CFP were incubated with zymosan and then fixed. The efficiency of phagocytosis was calculated. The results are expressed as a percentage of control (untransfected) cells. The means ± SEM of three independent experiments are plotted. Student's *t*-test was used for statistical analysis. ^*∗*^ *p* < 0.05 compared to CFP transfected cells.

Although ARF6 has been shown to be associated with phagosomal membranes and to regulate Fc*γ*R-mediated phagocytosis [38], it remains unclear whether or not ARF6 is involved in the uptake of zymosan particles. To investigate ARF6 dynamics during the stages of phagosome formation, the internalization of zymosan was observed by confocal laser microscopy in live RAW264 macrophages expressing ARF6-CFP. Prior to the onset of phagocytosis, ARF6 was observed in the cytosol, some intracellular vesicles, and the plasma membrane. After the addition of zymosan to the cells, ARF6-CFP was recruited to the plasma membrane of the phagocytic cups along the surface of zymosan particles (Figure 7(a), *t* = 3 min). The levels of ARF6 in the phagocytic membranes peaked at around three minutes after the zymosan bound to the cells. Subsequently, ARF6 was transiently localized on the membranes of newly formed phagosomes and dissociated from them over time (Figure 7(a); *t* = 6 min and 24 min, resp.).

To further confirm ARF6 involvement in phagocytosis of zymosan, we monitored the activation levels of endogenous ARF6 using the ARF binding domain of GGA3, which interacts with the GTP-bound form of ARF6 [39]. As shown in Figure 7(b), the activation of ARF6 was observed two minutes after the addition of zymosan particles. The GTP-bound form of ARF6 reached a maximum at around 2–5 minutes and then gradually returned to basal levels. These data indicate that endogenous ARF6 is transiently activated during an early stage of zymosan ingestion.

We further examined the effects of the expression of ARF6 mutants on zymosan internalization into phagosomes. RAW264 cells were transiently transfected with wild-type (wt) ARF6-CFP, GTP-bound mutant (active form) ARF6-Q67L-CFP, or GDP-bound mutant (inactive form) ARF6-T27N-CFP. Then, a quantitative assay of phagocytosis of zymosan was performed using cells expressing the ARF6 allele. As shown in Figure 7(c), the expression of either ARF6-Q67L or ARF6-T27N led to inhibition of the phagocytosis of zymosan, in contrast to the CFP control. These data indicated that the internalization of zymosan into phagosomes requires normal cycling of ARF6 between GTP- and GDP-bound forms.

## 4. Discussion

This study provides the first evidence that Rab35, ACAP2, and ARF6 are involved in phagocytosis of zymosan. Our live-cell imaging analysis showed that Rab35 is recruited to the membranes of phagocytic cups and then dissociated from the membrane of newly formed phagosomes. Importantly, the novel pull-down assay for GTP-Rab35 demonstrated that Rab35 is inactivated during phagosome formation. Moreover, we found that the expression of GTP-bound mutant Rab35-Q67L or GDP-bound mutant Rab35-S22N inhibits phagocytosis of zymosan. These data suggest that the activation-inactivation cycle of Rab35 is required for the uptake of zymosan. In our pull-down experiments with whole-cell lysate, we did not detect activation of Rab35 during the early stage of phagocytosis. It is assumed that a small amount of Rab35 in the cells may be locally activated at the initial stage of phagosome formation.

It is important to measure the GTP-Rab35 levels to investigate this protein's function. Previously, it has been reported that RBD35 (Rab-binding domain specific for Rab35) of RUSC2 traps the GTP-bound form of Rab35* in vitro* [40]. In this study, we found that a GST fusion protein containing the ANKR domain of ACAP2 interacts with the GTP-bound form of Rab35 but not of Rab21 or Rab11. We think that GST-ANKR of ACAP2 is also a useful tool to monitor the activation levels of Rab35. The ANKR trap of ACAP2 specifically recognizes switch II domain of Rab35, whereas the RUSC2 trap may recognize not only switch II domain, but also some other regions of Rab35 [41]. Because specific recognition and affinity are quite important as a tracer, further studies will be necessary to conclude which tracer is the best to monitor the GTP-bound form of Rab35.

Our data from time-lapse imaging revealed that ACAP2, a GAP for ARF6, is localized to the membranes of phagocytic cups and that the expression of Rab35 facilitates the recruitment of ACAP2 to the phagocytic membranes. The increase in the amount of ACAP2 in the plasma membranes correlated with the inhibitory effect on phagosome formation. In fact, while the expression of ACAP2 subtly inhibits phagocytosis of zymosan, the coexpression of ACAP2 and GTP-bound mutant Rab35-Q67L, which induces plasma membrane localization of ACAP2, has a remarkable inhibitory effect on the rate of phagocytosis. Since expression of the ARF6-GAP defective mutant of ACAP2 or the Rab35 binding-defective mutant of ACAP2 had no effect on the uptake of zymosan, it is suggested that the inhibitory effect of ACAP2 on phagocytosis requires the GAP activity of ACAP2 toward ARF6 and the binding of ACAP2 to Rab35. Interestingly, we found that the expression of plasma membrane-targeted ACAP2 alone downregulates ARF6 activity and impairs phagocytosis of zymosan to a similar extent to coexpression of ACAP2 and Rab35-Q67L. Previous reports have shown that the GTP-bound form of ARF6 is localized to the plasma membrane, whereas the GDP-bound form of ARF6 is found on intercellular endosomes [42, 43]. Altogether, our data support the notion that Rab35 mainly regulates plasma membrane targeting of ACAP2 in a GTP-Rab35-dependent manner and that ACAP2 functions predominantly in plasma membranes during phagosome formation.

During Fc*γ*R-mediated phagocytosis, ARF6 has been shown to be involved in phagosome formation [33, 38]. In this study, we also found that ARF6 is transiently activated and regulates the uptake of zymosan. Furthermore, the expression of the GTP-bound mutant ARF6-Q67L or GDP-bound mutant ARF6-T27N inhibits phagosome formation. These data indicate that the activation-inactivation cycling of ARF6, similarly to Rab35, is required for the uptake of zymosan.

Currently, the downstream molecule of ARF6 during zymosan phagocytosis remains unknown. It has been shown that phosphatidylinositol 4-phosphate 5-kinase (PI(4)P 5-kinase), a downstream effector of ARF6, regulates the uptake of* Chlamydia* and* Yersinia* [31, 32]. Based on these findings, it is postulated that ARF6 may regulate phagocytosis of zymosan via PI(4)P 5-kinase, facilitating the production of phosphatidylinositol 4,5-bisphosphate (PI(4,5)P_2_), which stimulates actin polymerization. Intriguingly, it has been shown that Rab35 interacts with the PI(4,5)P_2_ 5-phosphatase, Oculocerebrorenal Syndrome of Lowe Protein (OCRL), and remodels the actin cytoskeleton [44]. Furthermore, OCRL has been shown to be involved in phagocytosis of* Yersinia* and* Listeria* [45, 46]. Thus, ARF6 and Rab35 may act antagonistically in zymosan phagocytosis through the regulation of phosphoinositide metabolism.

The specific GAPs for Rab35 during zymosan phagocytosis are currently unknown. Recently, it was shown that the GTP-bound form of ARF6 interacts with EPI64B (a GAP for Rab35) and negatively modulates Rab35 activity in clathrin-coated structures [47]. As described above, we demonstrated that ARF6 is transiently activated and recruited to the phagocytic membranes during phagocytosis of zymosan. In addition, ARF6 and Rab35 are colocalized at the membrane of phagocytic cups (data not shown). Therefore, it is also conceivable that ARF6 may downregulate Rab35 activity through recruitment of EPI64B to the plasma membrane as a negative feedback mechanism.

Our study demonstrated that the inactivation-activation cycling of both Rab35 and ARF6 is required for the phagocytosis of zymosan. We also found that Rab35 is a crucial molecule that downregulates ARF6 signaling via ACAP2 recruitment during zymosan phagocytosis. In light of the previous report that ARF6 inhibits Rab35 activation, it is postulated that interplay between Rab35 and ARF6 may regulate zymosan phagocytosis via mutual antagonism. In future studies, not only the identification of specific GAPs for Rab35 but also the definition of guanine-nucleotide exchange factors (GEFs) for Rab35 and ARF6, which modulates and integrates Rab35/ARF6 signaling, would be required to understand the antagonistic molecular module during phagosome formation.
